# Undiluted 25% intralesional sodium thiosulfate in the management of dystrophic calcinosis cutis

**DOI:** 10.1016/j.jdcr.2024.07.014

**Published:** 2024-08-03

**Authors:** Katherine Benandi, Devon Sieving, Amy Bumgardner, Kristin Wolf

**Affiliations:** a Complete Dermatology, Conroe, Texas; b John Sealy School of Medicine, University of Texas Medical Branch, Galveston, Texas

**Keywords:** calcifying disorders, calcinosis cutis, dermatomyositis, sodium thiosulfate

## Introduction

Calcinosis cutis is a multifactorial condition defined by insoluble calcium salt deposition in cutaneous and subcutaneous tissue. There are 5 etiology-dependent subtypes associated with this condition, including dystrophic, metastatic, idiopathic, iatrogenic, and calciphylaxis. Dystrophic calcinosis cutis, the most common subtype, is associated with autoimmune connective tissue disease, including dermatomyositis.^1^

Management of calcinosis cutis is often challenging, as treatments are subtype dependent and vary with factors such as size and severity. It is particularly difficult to treat dystrophic calcinosis cutis that is secondary to autoimmune connective tissue disease. The primary goal is often related to improving quality of life, rather than total resolution of the lesion.^2^ Treatment options include various pharmacologic therapies as well as surgical resection, although it remains unclear which is most effective.

One emerging pharmacologic option for dystrophic calcinosis cutis is topical or intralesional sodium thiosulfate (STS). Existing reports of intralesional STS therapy have demonstrated variable success rates. In a double-blind placebo-controlled pilot study where patients underwent 12 weeks of monthly injections of STS 40 mg/mL, only 1 of the 4 patients who completed therapy had a positive result.^3^ Another study in Germany reported 100% total resolution in 13 patients with calcinosis cutis with lesions ≤2 cm treated with 250 mg/mL (undiluted) STS.^4^ The same study cited a failure to fully resolve any lesions >2 cm when using this method.^4^ It is unknown, however, whether these patients experienced any symptomatic improvements, especially those related to quality of life.

Adverse effects associated with intralesional STS include injection site pain/irritation and ulceration, which may be exacerbated at higher concentrations.^5^ This often results in treatment at diluted concentrations, which may be less efficacious, especially when treating larger lesions. This case describes a patient with a dystrophic calcinosis cutis lesion who experienced significant improvement with a series of undiluted intralesional STS injections.

## Case report

**Fig 1:**
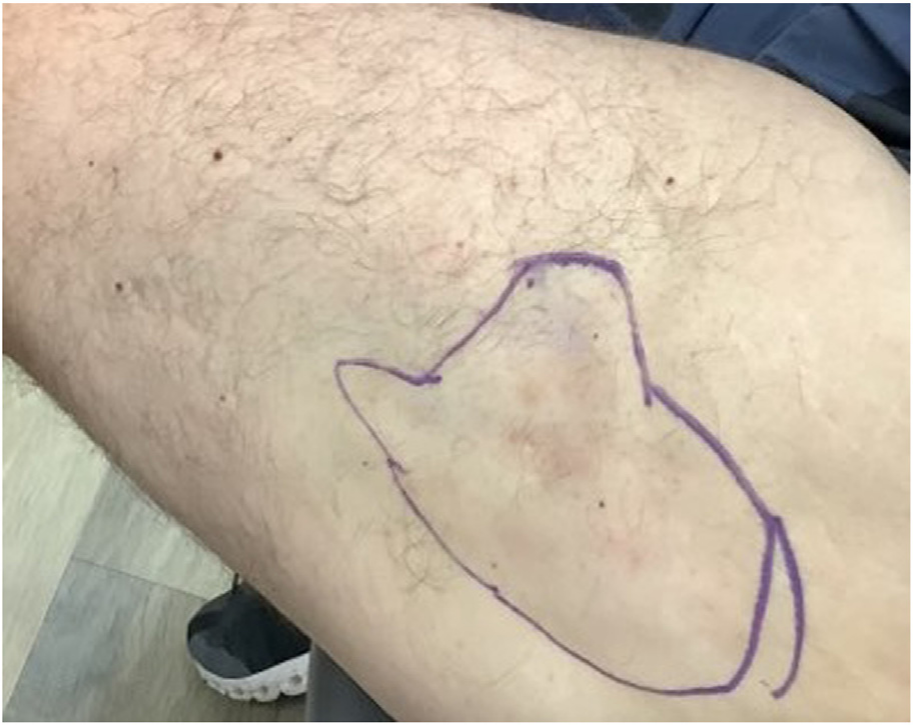
Calcinosis cutis lesion measuring 11 × 9 cm at initial presentation. Estimated margins of the lesion are marked by the *encircled area*.

A man in his early 50s with a history of dermatomyositis presented with an 11 × 9 cm indurated, white cutaneous, painful nodule on lateral aspect of the upper portion of his left thigh (Fig 1). Subsequent biopsy revealed large fragments of calcification of the subcutis without evidence of vasculitis or malignant/atypical features, consistent with a diagnosis of dystrophic calcinosis cutis secondary to dermatomyositis. Over the course of a year, the severity of the lesion fluctuated with dermatomyositis control, as the patient attempted various therapeutics including azathioprine and mycophenolate mofetil.

After lack of improvement with mycophenolate mofetil and increasing discomfort associated with the lesion, the patient elected to begin intralesional STS therapy. Of note, the patient did not have renal impairment prompting possible dose adjustment. Before all STS treatments, the area was infiltrated with lidocaine 1% solution to make injections more tolerable. The first treatment consisted of 2 different concentrations, which served to compare both efficacy and dose-dependent side effects. The anterior aspect of the lesion was injected with 1 mL of 150 mg/mL, whereas the posterior aspect was injected with 0.3 mL of 250 mg/mL, undiluted STS. The patient denied significant differences in pain upon injection with different concentrations. At 2 week follow-up, he did not show significant improvement, but reported no adverse effects with either concentration. Because the patient was able to tolerate the higher concentration well, all subsequent treatments were performed with 250 mg/mL undiluted STS, in an attempt to maximize therapeutic benefit.

**Fig 2:**
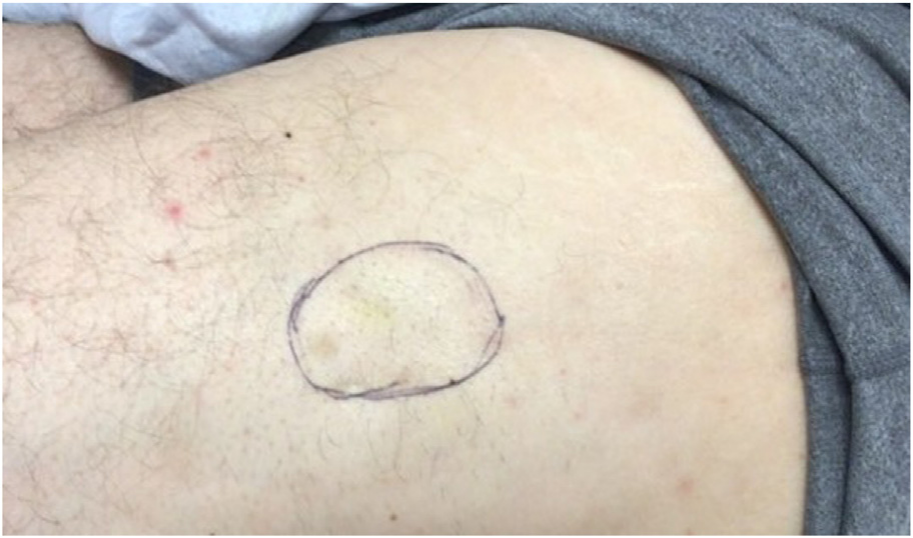
Calcinosis cutis lesion measuring 5 × 4 cm after 12 intralesional sodium thiosulfate treatments over a 13 month period. Estimated margins of the lesion are marked by the *encircled area*.

The patient underwent 11 additional treatments in 2 to 6 week intervals over the span of 13 months, ranging from 2 to 6 mL of undiluted STS. He tolerated each treatment well with no ulceration or other adverse effects noted. The patient first reported mild improvement after his second treatment, noting the lesion had both softened and decreased in size at 3 week follow-up. He continued to progress throughout treatment duration, ultimately reporting less pain and discomfort associated with the lesion. The final size of the lesion was 5 × 4 cm (Fig 2).

## Discussion

This is the first report of significant improvement of a large, dystrophic calcinosis cutis lesion from undiluted 25% STS therapy. As such, it highlights the importance of reporting qualitative data such as symptomatic improvement and enhanced quality of life as measures of success in treating this condition. Prior studies, which only assess success by total resolution, may fail to recognize what would otherwise be classified as substantial improvement for a patient with this complex condition. This was the case for our patient, who noted significant reduction of pain and discomfort associated with his lesion, despite lack of total clearance.

Although we lack data on efficacy for lower concentrations of STS versus undiluted STS, our findings highlight the potential clinical utility of undiluted STS in the management of dystrophic calcinosis cutis. Additionally, the patient’s lack of severe injection site pain or ulceration warrant further investigation into the potential for routine use of higher concentrations of STS in this condition. If it proves to be equally tolerable and more efficacious than previously attempted concentrations, patients may achieve faster therapeutic effects or heightened levels of overall improvement.

## Conflicts of interest

None disclosed.
